# Evaluation of occupational exposure to different levels of mixed organic solvents and cognitive function in the painting unit of an automotive industry

**DOI:** 10.15171/hpp.2018.42

**Published:** 2018-10-27

**Authors:** Farideh Golbabaei, Fateme Dehghani, Mohammad Saatchi, Seyed Abolfazl Zakerian

**Affiliations:** ^1^Department of Occupational Health Engineering, School of Public Health, Tehran University of Medical Sciences, Tehran, Iran; ^2^Department of Biostatistics and Epidemiology, School of Public Health, Hamadan University of Medical Sciences, Hamadan, Iran

**Keywords:** Automotive industry, Cognitive function, Occupational exposure, Painting unit, Volatile organic compounds (VOCs)

## Abstract

**Background:** The cognitive function could be affected following exposure to organic solvents.The purpose of this study was to examine the cognitive performance of workers exposed to different levels of organic solvents in a painting unit of an automotive industry.

**Methods:** This case-control study was conducted, in 2017, on 121 and 111 workers from the painting and assembly units of an automotive industry as the case and control groups,respectively. Exposure of workers to organic solvents were determined according to National Institute for Occupational Safety and Health (NIOSH) method. The cognitive performance of the studied population was evaluated by the computerized tests.

**Results:** The obtained scores of the N-Back in 1 and 2 blocks and the simple reaction time tests(SRTTs) were significantly differed in the exposed group (p<0.05). No significant difference was observed between case and control groups in the Stroop test score (p> 0.05). Moreover, a significant relationship between the hazard quotient and the cognitive function test scores were observed except the Stroop test.

**Conclusion:** Exposure to organic solvents affect the cognitive functions even below the Occupational Exposure Limits (OELs). Moreover, workers with high exposure levels of organic solvents had highest risk of poor memory and reaction time.

## Introduction

Broad range applications of volatile organic compounds (VOCs) such as degreaser and constituents of ink, paint, aerosol spray and intermediates has led to exposure of workers to different organic solvents.^1^ Lipophilicity and volatility as the 2 important properties of organic solvents help their absorption by cellular membrane and lipid-rich tissues such as brain organ. These characteristics produce adverse effects on the central nervous system (CNS) in acute-high level exposure.^2,3^ Although the neurotoxic effects of solvents on the peripheral nervous system in chronic-moderate and high-level exposures have long been well documented, persistent effects of long-term and low-level exposures on the CNS have remained unclear.^4^ Alteration in domains of memory, attention, and other cognitive functions status as neuropsychological endpoints are linked with organic solvent exposure.^5^ Various studies have been carried out to investigate adverse effects of chronic low-level exposure on cognitive functions.^6,7^ In the majority of studies, the exposure assessment parameters have not been clearly considered. For example, some studies have only investigated the cognitive effects of exposure to one particular solvent. Considering the combined effects of solvent exposure is of great importance because it is well known that organic solvents are usually present in mixed form at work places.^8,9^ Therefore, applying a suitable indicator to consider effects of mixed solvent exposure is necessary.^10^ An exposure index (EI) has been widely used in order to estimate the occupational exposure to mixed organic solvents in previous studies. EI is calculated based on the ratio of the concentration of solvents to the threshold limit value.^7^ Whereas, it is well-known that the endpoints taken into account for the determination of threshold limit values are rarely based on the neurotoxic effects.^10^ Additionally, it is well recognized that cognitive performance is directly influenced by duration of exposure and organic solvent concentration. In the previous human studies, the different levels of exposure as the main factor in cognitive decline have rarely been considered. Furthermore, conducting animal studies that take into account different ranges of exposure to VOCs is almost difficult and needs a large budget. Based on the above-mentioned descriptions, it is required to establish a dose-response relationship between the levels of exposure to organic solvents and the cognitive function decrease.

To the best of our knowledge, there is no study to consider the relationship between different exposure levels of mixed organic solvents and neurotoxic effects. Giving to the aforementioned issues, this study was aimed at evaluating the cognitive performance and mixed organic solvents exposure using a new approach.

## Materials and Methods

### Site and participant recruitment

This case-control study was conducted on a total of 232 male participants, including 121 cases and 111 controls, in the morning shift of the painting and assembly line units in an automotive industry in Tehran, Iran in 2017. The painting unit workers had been exposed to organic solvents through different painting processes and the assembly line workers had no history of solvent exposure. In addition, the control group workers had no significant exposure to the confounding factors such as noise and heat stress. To examine the dose-effect relationship, painting unit workers were divided into 3 groups including spray painters, pre-painters and polishing workers.

### Data collection

*Background information*

Personal and occupational information was obtained using a self-designed questionnaire. Informational on work characteristic such as employment date, job history, work shift schedule, working hours per day and use of personal protective equipment was collected. All potential personal confounders including, level of education, personal habits such as alcohol and caffeine consumption, smoking, use of medication, drug abuse and also other following confounders including cerebral or head injury, hypertension, and exposure to neurotoxic solvents during leisure time were inquired about, asked and recorded from participants.

### Solvent exposure assessment

In order to identify the type of solvents in the case group workers and also being sure that control group workers have no exposure to organic solvents, four samples (2 samples from each group’s workplace) were collected from the workplace air. In the next step, the collected samples were analyzed by a gas chromatography-mass spectrometry (GC-MS) (CP-3800, Saturn-2200- VARIAN). The results of the GC-MS analysis indicated that no chemical substances were detected in the workplace air of the control group (the group with no history of exposure to organic solvents). Moreover, analyzing the samples taken from workplace air of the exposed group revealed that benzene, toluene, ethylbenzene, and xylene (BTEX) are the most significant organic solvents involved with this group. In the next step, the NIOSH 1501 analytical method was used for sampling and analysis of BTEX compounds. The number of required samples for exposure assessment of exposed group to BTEX compounds was estimated to be 36. To conduct sampling, similar exposure groups (SEGs) were determined and the required samples (36 samples) were assigned to the three exposed groups including spray workers, pre-painting and polishing workers. Then, the breathing zone air of the workers was sampled on charcoal tubes (SKC 226-01) using a portable micro pump. The pump flow rate was adjusted to 50-200 mL/min. After sampling, the samples were immediately transferred to the lab. Extraction of the samples was carried out, using 1.0 mL of carbon disulfide (99.5%) (Merck, Germany) as the eluent. After extraction, the samples were analyzed through Gas Chromatography using a Flame Ionization Detector (GC-FID) (Varian, CP3800).

The EI was used to evaluate the combined effects of current exposure to organic compounds and it was calculated using the following equation^11^:

$$EI\;=\;\frac{C_{1}}{OEL_{1}}\;+\;\frac{C_{2}}{OEL_{2}}\;+\;\frac{C_{n}}{OEL_{n}}$$

Where, *C* is the concentration of organic solvents, and *OEL* is occupational exposure limit based on international standards.

### Cumulative exposure as a result of combined exposure

The average inhalation exposure over the workers’ lifetime was calculated through the average daily inhalation intake based on the annual average exposure level:

$$I\;=\;\frac{C\times \;ET\times EF\times ED}{AT}$$

Where *I* is defined as the average daily inhalation intake (μg/m^3^), *C* is the concentration of the compound in the breathing zone air of the workers (μg/m^3^), *ET* is the exposure time (h/day), *EF* is the exposure frequency (days/year), *ED* is the exposure duration (years), and *AT* is an average lifetime (hours).

In order to assess cumulative and lifetime relative risk, the hazard quotient index (HQ) was utilized.^12^ Using to the following equation, cumulative exposure to organic solvents was calculated for each subject.

$$HQ=\frac{I}{MRL}$$

The MRL is the minimal risk level introduced by ATDSR.^13^

### Cognitive testing 

Three domains of neurobehavioral functions including psychomotor speed, memory, and attention, which have been identified as the factors affected in occupational exposure to organic solvents, were measured using the computer-controlled tests. In order to eliminate the effects of any distracting factors, all the tests were administered and conducted in a quiet room using a personal computer.

### N-back test

The computerized version of the n-back test was applied to assess memory function by increasing task load. Working memory was assessed through n-back 1 and 2 tests.^14^ The n-back test is a continuous performance task that is commonly used to measure a part of working memory and working memory capacity. To conduct the test, a sequence of 100 numbers as stimuli was presented for each participant. The participants were requested to memorize the recently presented stimulus and decided whether it matches the one n steps back in the sequence. The load factor “N” can be adjusted to make the task more or less difficult. For example “1-N” means that participants have to remember the position of the item one turn back and “2-N” means remembering the position of the item 2 turns back, and so on.^14^

### Stroop test

Selective attention, the ability to ignore distracting visual and verbal stimuli, was measured by the computerized Stroop test. In this task, a number of 4 circles in different colors (blue, red, green and yellow) were presented on the computer screen. The name of the color which might be conflicted with its color had been written. The participants were asked to select the right button on the keyboard according to the presented color not written color names.^15^

### Reaction time test

Vasomotor speed was measured by a computerized simple reaction time test (SRTT). In this test, the workers were instructed and requested to react as quickly as they can recognize the 50 identical optical signals by pressing the selective button on the key board.^16^

### Statistical analysis

Statistical analyzing was performed by using SPSS software (version 22). A *P* value < 0.050 was considered as a significant level within the 95% CI.

The paired student^’^s *t* test was used to examine significant differences between case and control groups. Analysis of variance (ANOVA) was also used to compare variables among multiple groups. Moreover, the multiple adjusted regressions were applied to find out the relationship between participant characteristics such as age, working experience, smoking habit as well as hazard quotient and cognitive test scores.

## Results

In total, 32 subjects could not to meet inclusion criteria and were excluded from the analysis. It is worth mentioning that lack of education as the main factor contributed to decreased cognitive performance was controlled through limitation. In the other words, all participants had mid-level of education and a few with different educational status were excluded from the study. Also, the subjects with alcohol consumption were excluded from analysis. Actually, there was an uncertainty in worker’s report on alcohol usage and therefore, we were unable to establish a correct statistical analysis for the alcohol usage content and cognitive performance. Finally, data was provided for 200 workers, including 98 painting unit workers (exposed group) and 102 reference workers (control group). Table 1 shows the distribution of demographic characteristics within different groups (exposed and non-exposed groups). There were no significant statistical differences between exposed and non-exposed workers in age, work experience, and smoking status. In the exposed group, the appreciable difference was found between spray and polishing workers in work experience.

### Exposure assessment 

After analyzing the samples gathered from different sections of the breathing zone of painting unit workers, the BTEX compounds were recognized as the main organic solvents according to GC-MS analysis results. The average and standard deviation of organic solvent concentration as well as the EI have been presented in Table 2. According to data provided in Table 2, the concentration of BTEX in the spray workers section was much higher than other studied groups and benzene exposure in this section was higher than the OELs (the OELs for benzene is 0.5 ppm). Among the analyzed sections, the polishing workers section had the least level of exposure to BTEX compounds. The obtained concentrations of BTEX compounds for the polishing workers section was far below the OEL. In addition to the individual concentrations of the BTEX compounds, the mixed organic solvent expo sure level, which is expressed through an EI, was much higher in the spray workers group than other analyzed groups; this level was also higher than the values provided by international exposure standards.^17^ It is worth mentioning that the calculated EI for polishing workers group was far below 1.

Table 3 summarizes the hazard quotient levels in the exposed group. The Hazard quotient which is used in neurological risk assessment research was applied to assess the individual cumulative exposure level.^12^ As mentioned before, the hazard quotient is obtained from inhalation intake divided by the minimal risk level (MRL); MRL amounts for BTEX are 0.003, 1, 0.06 and 0.05, respectively. The affecting parameters for calculation of inhalation intake are exposure level, age and work duration, which influence on the neurobehavioral functions. Using the hazard quotient, it is possible to compare the neurobehavioral status of workers with different working characteristics. As it is seen, the hazard quotient level in spray workers (painters) is higher than the others.

### Cognitive functions

Table 4 shows the cognitive functions and mood status among different sub-groups of exposed workers. The significant differences were found in memory status in both 1 and 2 blocks of the n-back test as well as the SRTT. The memory function score was lower in the exposed workers in comparison to the unexposed groups. The reaction time was also clearly longer in exposed workers than the control group. According to the presented data in Table 4, no significant differences were observed in the Stroop test between the case and control groups.

Bivariate correlation coefficients showed a significant relationship between the hazard quotient and test scores except for Stroop test which did not change with the HQ value (Table 5). As Table 5 indicates, there was a positive correlation between the hazard quotient and the reaction time test. Furthermore, the correlation between the n-back tests (n-back 1 and 2) and the hazard quotient was positively significant.

Moreover, multiple regression analysis did not show any significant relationship between the cognitive tests and the demographic data except for one between the duration of exposure and the SRTT (data was not shown).

## Discussion

This study investigated the occupational exposure to different levels of mixed organic solvents and its effects on the cognitive functions of the workers in the painting unit of an automotive industry. To the best of our knowledge, our study was the first study which investigated the effects of exposure to mixed BTEX compounds on cognitive performance. In order to identify the types of organic solvents in the exposed group (painting unit), environmental samples were taken from the painting unit and subsequently were analyzed by GC-MS. Based on obtained results from GC-MS analysis, BTEX compounds, which neurotoxicity effects have been confirmed in previous studies,^18-20^ were selected as the selective solvents in the present study. In addition to the neurological characteristics, the peak level of BTEX compounds was much bigger than the other identified compounds in the GC-MS chromatogram. Therefore, BTEX compounds were selected as the main compounds for further experiments. According to the Koffi Badjagbo study, these compounds have also been reported as the main organic solvents emitted from the automotive industry.^21^ Two important factors affecting cognitive functions in occupational exposure to organic solvents, which have been reported in the previous study, are level and duration of exposure.^10,22^

Although the concentration of toluene, ethylbenzene, and xylene in the spray workers group was lower than the occupational exposure level (OEL), the cumulative EI of BTEX was higher than the provided standard value. This finding emphasizes that benzene concentration has a substantial effect on increasing the combined EI value concerning spray workers. Regarding the obtained results, the spray section was known as the most polluted environment among the studied sections. This finding is in line with the Dehghani et al study.^23^ The possible reasons for the high level of benzene in this section may be the presence of benzene as an impurity in solvents and also the unnecessary use of thinner for cleaning the surface. Cognitive test scores revealed that spray workers (painters) had a poorer cognitive function in the domain of memory and reaction time. There has been a long history of research related to neurotoxicity of solvents in painters.^7,24^ Memory has been stated as the most prevalent domain of cognitive performance alteration in occupational exposure to mixed organic solvents.^1,25^ In this study, there was a noticeable difference between the exposed and the control group in the reaction time test. This finding is in line with most previous similar studies.^7,26^ Although in a meta-analysis study, conducted by Meyer-Baron, attention was introduced as the most frequent domain of cognition deterioration in occupational exposure to organic solvents, in our study, there were no significant differences in the Stroop test scores between the case and control groups. Several reasons might explain this inconsistency in the findings across these studies. In the present study, toluene, which is well-known to have more neurotoxic characteristics than the other studied compounds (benzene, ethylbenzene, and xylene), had the lowest concentration. It is worth mentioning that in the Meyer meta-analysis, the average concentration of toluene, which was reported to be responsible for altering the attention was in the ranges of 33 to 89 ppm,^10^ while in the current study, the concentration of toluene was much lower than the reported value of the Meyer research. The second reason for our findings is attributed to the type of the test used for evaluating the attention status. In the present study, a computerized test was used to investigate the attention status.

All 3 exposure groups had a greater risk of poorer memory in both of the 1 and 2 blocks of n-back compared to the reference group. Although polishing workers had a trivial exposure level to organic solvents, they got a lower memory score than the non-exposed workers. This finding indicated that occupational exposure to low levels of organic solvents can also affect memory status in long-term exposures.

As presented in Table 5, cognitive test scores also changed as the hazard quotient values increased. In the hazard quotient equation, three important parameters which are age, working experience and level of exposure were considered. Given that there was not a significant difference between age and working experience (*P* value >0.05), the level of exposure was regarded as the main variable for calculating the hazard quotient value. Given all the above, it seems that exposure level plays an important role in cognitive performance alteration. Moreover, in a cohort study, exposure to high levels of organic solvents proved to have resulted in decreasing cognitive functions.^22^ These findings were in line with our study results. The results of the other study, which was carried out on the painters, revealed that the cumulative exposure level is directly associated with the alteration in cognitive performance.^27^

## Conclusion

In spite of the call to remove benzene from the paint ingredients, it still seems to be a matter in the workplace. Exposure to the low levels of the BTEX compounds, even below the OELs, has caused an increased risk of neurobehavioral changes in the domains of memory and reaction. Furthermore, increasing concentration levels and long-term exposure to organic solvents were significantly related to decrease in cognitive function scores. It is worth mentioning that hazard quotient, a quotient obtained by considering some potential factors that might affect neurobehavioral functions, showed a dose-response relationship in exposed workers in memory and especially in the reaction time tests.

## Ethical approval

To conduct this research, permission was obtained from the ethical committee of Tehran University of Medical Sciences (TUMS). Informed consent was also obtained from each participant.

## Competing interests

The authors declare that they have no competing interests.

## Funding

This study was conducted with financial support from the Tehran University of Medical Sciences.

## Authors’ contributions

FD collected the data and drafted the manuscript. FG and SAZ oversaw the whole study process and designed the study. MS performed statistical analysis. All authors read and approved the final manuscript.

## Acknowledgments

The authors would like to appreciate from Tehran University of Medical Sciences. We also appreciate the workers and HSE personal of the car manufacturing factory for their help and cooperation in this project.

**Table 1:** Demographic and work characteristics of the studied population

	**Spray worker (n = 22)**		**Pre-painting (n = 35)**		**Polishing workers (n = 45)**		**All painting workers (n = 102)**		**Reference workers** **(n = 98)**		***P*** ** value**
	**No.**	**%**	**No.**	**%**	**No.**	**%**	**No.**	**%**	**No.**	**%**	
Age-group											0.07
<30	2	5.7	0	0	0	0	2	2	5	5.1	
30-39	16	72.6	20	56.3	25	55.4	61	60	60	61.2	
40-49	4	18.18	14	40.6	15	33.3	33	32.3	30	30.6	
>49	0	0	1	3.1	5	11.11	6	5.8	3	3.1	
Smoking status											0.15
Non-smoker	11	50	13	36.4	11	29.4	35	37.5	33	32.5	
Current smoker	11	50	22	63.6	34	70.6	67	62.5	65	67.5	
Duration of employment (y)											0.36
<5	2	9	3	8.6	0	0	5	4.9	3	3.1	
5-10	6	27.1	3	8.6	6	13.3	12	11.7	15	15.3	
10-15	9	33.3	16	45.7	24	53.3	54	52.9	57	58.2	
15-20	5	22.7	13	37.1	15	33.3	31	30.4	22	22.4	
>20	0	0	0	0	0	0	0	0	1	1	
Work hours per day											0.6
<8	0	0	0	0	0	0	0	0	0	0	
8-11	18	82	33	94.3	40	89	91	89	90	92	
>11	4	18	2	5.7	5	11	11	10.8	8	8.2	

**Table 2:** Solvent exposure levels in painting section (ppm)

**Solvent**	**Spray workers**	**Pre-painters**	**Polishing workers**
	**Mean ± SD**	**Mean ± SD**	**Mean ± SD**
Benzene	0.96 ± 0.05	0.33 ± 0.12	0.18 ± 0.08
Toluene	0.28 ± 0.08	0.14 ± 0.076	0.03 ± 0.014
Ethylbenzene	2 ± 0.2	1.44 ± 0.65	0.27 ± 0.1
Xylene	3.02 ± 0.08	1.85 ± 0.87	0.94 ± 0.04
EI	2.6 ± 0.88	0.76 ± 0.23	0.27 ± 0.12

Abbreviation: EI, exposure index.

**Table 3:** Hazard quotient levels in different sub-groups of exposed group

**Solvent**	**Spray workers**	**Pre-painters**	**Polishing workers**
	**Mean ± SD**	**Mean ± SD**	**Mean ± SD**
Benzene	37.5± 1.96	13.7 ± 4.34	4.9 ± 0.92
Toluene	3 ± 0.98	1± 0.12	2.4 ± 0.9
Ethylbenzene	4.1 ± 1.12	2.8 ± 0.67	0.54 ± 0.11
Xylene	7.07 ± 1.2	4.4 ± 0.98	2.2 ±0.44

**Table 4:** Cognitive functions and mood test scores in exposed and unexposed workers

**Test**	**Exposed**				**Unexposed**	***P*** ** value**
	**Spray workers** **(n= 22)**	**Polishing workers** **(n=35 )**	**Pre-painting workers** ** (n=45)**	**All painting workers** **(n=102)**	**Reference workers (n=98)**	
Memory type						
n-back 1	95.74 ± 15.4	102.8 ± 16.4	103.2 ± 1.2	100 ± 17.2	108 ± 11.2	0.030
n- back 2	75 ± 16.04	81.7 ± 11.8	85.2 ± 18.36	80.6 ±19.2	90.5 ± 8.4	0.050
Psychomotor speed						
Simple reaction time (ms)	427.07 ± 10.07	405.7 ± 96.1	398.8 ± 75.1	409 ± 100.1	352 ± 50.3	0.003
Attention						
Visual Stroop test	0.97 ± 0.3	0.93 ± 0.49	0.88 ± 0.18	0.92 ± 0.5	1.07 ± 0.23	0.150

**Table 5:** Bivariate Correlation coefficients between hazard quotient and neurobehavioral tests

**Test**	**Hazard quotient**	
	***P*** ** value**	**Correlation**
n-back1	<05	- 0.55
n-back 2	<05	- 0.68
Reaction time	0.02	0.27
Attention	0.6	0.04
